# Nursing in India

**Published:** 1922-11

**Authors:** 


### NURSING IN INDIA.

THE nursing profession in India, as in other countries, is in a state of transition. In no part of the world is the changed outlook of the whole profession more noticeable. In every province of India more openings are being found for the trained nurse, and although much progress has been made during the past ten years in the training of nurses, the supply is by no means equal to the demand. It is not uncommon, even in the large towns, to find no trained nurse of any kind available, and women with little or no training have frequently to be employed to nurse serious cases. Many of the hospitals are very understaffed, and in most of the civil hospitals in the country districts no women nurses are employed.

A great desire for improvement in this direction is found all over India, and some of the Provincial medical administrations who realise the importance of employing trained women nurses in the hospitals of the towns are endeavouring to organise what may be called Provincial nursing services. If such services were established a definite curriculum would be laid down in the training schools. The nurses would become members of the service from the day they entered the hospital, they would be required to pass a central qualifying examination at the end of their service, and would then be drafted to the various hospitals in the Province. The grades in the Provincial nursing services would be matrons, sisters, staff nurses and probationers, with suitable pay and allowances for each grade.

If these schemes are brought to a successful conclusion they will undoubtedly greatly stimulate recruiting for the profession of nursing, for a member of such a service will be assured of good pay and prospects, possibly a pension, and will have to be a thoroughly trained woman of a good standard. It is possible that at first Indian girls would be trained only for work in women's and children's hospitals, as the prejudice against allowing their daughters to enter hospitals where men patients are nursed is still extremely strong among parents of good class Indian girls.

It is the earnest hope of all who are interested in nursing in India that the establishment of such nursing services will be inevitably followed by a scheme for the registration of nurses on lines applicable to the special needs of India and Burma. The Trained Nurses' Association of India is working steadily towards this end, and is endeavouring in every possible way to enlist the sympathy and interest of the medical profession.

European nurses play a great part in the training of Anglo-Indian and Indian nurses, and trained British sisters are found in almost every large hospital, engaged in the teaching of probationers and the inculcating of English methods wherever possible. Most of the Government hospitals in large towns employ English trained sisters, who usually have the supervision of wards containing up to 70 or 90 patients. An Englishwoman coming out to India for work in a general hospital is confronted with many difficulties, perhaps the greatest being the learning of the language, or rather languages, of the district in which she works; the absolutely different outlook and ideas of life with which she is confronted, and the either childlike trust or uneasy suspicion with which her patients will regard her. The Indians are almost always grateful patients, but they are timid, and inclined to doubt English methods, so different from their own deep-rooted traditions. It is in nursing Indian patients that the Mission nurse usually succeeds, and it is not too much to say that every nurse coming to India for work in hospital should be imbued with a missionary spirit which will enable her to see the best side of her work and to retain the love for her patients which she should have learnt in her probationer days.

The pay of an English nurse coming to India to work as a sister in a large hospital is usually somewhere about Rs.130 to Rs.200 a month, with suitable allowances for diet, uniform and washing. Most Government hospitals have provident funds, to which the sisters and nurses subscribe 10 per cent. (or more) of their monthly salaries, a bonus of 50 per cent. to 100 per cent. being given on provident fund savings at the end of the period of service. The contract is usually for three or five years, and the cost of the passage is almost always paid in the first instance. Off-duty time is given on a liberal scale in India, generally four or six hours a day, or alternative "half days" or "mornings off." The pay of an Army sister is, generally speaking, higher than that of sisters in civil employ, but the expenses of messing, &c., in the various stations differs very greatly, and India is no longer a cheap place in which to live.

The best-known associations for private nursing are the Lady Minto's Indian Nursing Association, Home Secretary, Miss Ray, 54 Ashburnham Mansions, Chelsea, S.W.10, for India and Burma; and the Lady Ampthill Nursing Institute, Madras. Nurses are recruited from England and the Dominions for both these organisations for a term of three or five years. Passage and travelling allowances are paid on both outward and homeward voyage, and a salary of about Rs.110, rising to Rs.155, is given. In both Associations everything is provided, and the nurses have no expenses except for their personal needs. The Lady Minto's Association supplies matrons and sisters to various hospitals and nursing homes, both in the Hills and on the Plains, which enables the sisters to have a change from private nursing.

The greatest need in the nursing profession in India is a large number of women capable of training Indian probationers. Indian girls have very many of the characteristics which go to make good nurses; they are gentle, sympathetic, affectionate and obedient, but they depend absolutely on the sister or nurse immediately above them; they have little initiative and lack a sense of responsibility. They require a constant, sympathetic supervision, which can only be given by well-trained women who have both the time and the inclination to train their nurses in all the methods which they themselves employ, and have a fixed ideal of what a good nurse ought to be. The best type of British nurse for India is one who can both work and play well; one who is not likely to be dismayed at difficulties, and who can patiently teach the same things many times without losing her temper; who will appreciate the many social pleasures such as dances, tennis, parties, &c., which can be enjoyed in most Indian stations, but will retain the love of her work and maintain the dignity of her profession.

Another field of activity for the nurse is gradually being opened up in India in the form of health work. A school for the training of health visitors was opened in Delhi in 1918, and is steadily gaining the confidence of the educated classes. The students must be trained midwives and undergo a course of training lasting one year in subjects relating to health and sanitation, with course of lectures on home nursing, first aid, tropical diseases, &c. The students who pass the higher or "A" grade examination are eligible for appointments (usually under municipalities) as health and maternity supervisors at a good rate of pay, and those students who pass a less difficult examination and are awarded only a "B" grade certificate are appointed as assistants. Both Anglo-Indians and Indians are now working in the crowded cities of India, supervising the work of the *dais* (Indian midwives), establishing baby welcomes and classes for mothers, and spreading new light of hope and knowledge in Indian homes.

